# No increased risk of glucose metabolism disorders in adults with growth hormone deficiency undergoing long-term treatment with biosimilar somatropin (Omnitrope®): data from an observational, longitudinal study

**DOI:** 10.1186/s12902-019-0464-2

**Published:** 2019-12-11

**Authors:** Paolo Beck-Peccoz, Charlotte Höybye, Robert D. Murray, Suat Simsek, Markus Zabransky, Hichem Zouater, Günter Stalla

**Affiliations:** 1 University of Milan, Via Pietro Custodi 16, 20136 Milan, Italy; 2 Department of Endocrinology, Karolinska University Hospital, and Department of Molecular Medicine and Surgery, Karolinska Institute, 171 76 Stockholm, Sweden; 3 St James’s University Hospital, Beckett Wing, Beckett Street, Leeds, LS9 7TF UK; 4 Northwest Clinics, Wilhelminalaan 12, 1815 JD Alkmaar, Netherlands; 5 Sandoz Biopharmaceutical, c/o HEXAL AG, Industriestr. 25, D-83607 Holzkirchen, Germany; 6 Medicover Neuroendokrinologie, Orleansplatz 3, 81667 Munich, Germany; 7 Medizinische Klinik und Poliklinik IV der Ludwig-Maximilians-Universität, Ziemssenstr. 1, 80336 Munich, Germany

**Keywords:** Adults, Biosimilars, Diabetes mellitus, Growth hormone, Growth hormone deficiency, Omnitrope®

## Abstract

**Background:**

To evaluate the impact of treatment with recombinant human growth hormone (rhGH; Omnitrope®) on the risk of diabetes mellitus in adults with growth hormone deficiency (GHD), using data from the ongoing PATRO Adults post-marketing surveillance study.

**Methods:**

PATRO Adults is an ongoing post-marketing surveillance study being conducted in hospitals and specialized endocrinology clinics across Europe. All enrolled patients who receive ≥1 dose of Omnitrope® are included in the safety population. Patient profiles, containing all available study database information for each specific patient, were generated for all patients with adverse events (AEs) of diabetes mellitus while participating in the study. Diabetes mellitus was confirmed if fasting plasma glucose was ≥7.0 mmol/L or 2-h plasma glucose ≥11.1 mmol/L during oral glucose tolerance test or glycated hemoglobin ≥6.5%.

**Results:**

Up to July 2018, 1293 patients had been enrolled in the study, and 983 (76.0%) remained active. Just under half (*n =* 687, 49.3%) of the patients were growth hormone (GH) treatment-naïve on entering the study, and most (*n =* 1128, 87.2%) had multiple pituitary hormone deficiency (MPHD). Diabetes mellitus/inadequate control (worsening) of diabetes mellitus was reported in 21 patients (22 events). The cases were newly diagnosed in 15 patients (age 29–84 years; incidence rate 3.61 per 1000 patient-years) and occurred in 6 patients with pre-existing diabetes mellitus at baseline (age 45–72 years). Most cases of newly diagnosed diabetes mellitus occurred in patients with adult-onset MPHD (*n* = 13); the remaining cases of new-onset diabetes mellitus occurred in a patient with childhood-onset MPHD who had previously received GH replacement therapy (*n =* 1), and a patient with adulthood-onset isolated GHD who was naïve to GH replacement therapy (*n =* 1). All cases of inadequate control/worsening of diabetes mellitus occurred in patients with adult-onset MPHD*.*

**Conclusions:**

Based on this snapshot of data from PATRO Adults, Omnitrope® treatment is tolerated in adult patients with GHD in a real-life clinical practice setting. No signals of an increased risk for diabetes mellitus have been noted so far, although continued follow-up (both during and after rhGH therapy) is required to confirm this.

**Trial registration:**

Not applicable.

## Background

Growth hormone deficiency (GHD) is a well-recognized condition in adults, with an estimated prevalence of 2–3 per 10,000 population [1]. GHD in adults is typically characterized by detrimental effects on body composition, including abdominal obesity, decreased muscle mass and reduced skeletal muscle strength [2]. In addition, adult GHD has been associated with impaired lipid and carbohydrate metabolism, and an increased risk of cardiovascular complications, partly due to increased visceral obesity [3, 4]. The quality of life (QoL) in adults with GHD is also affected, with patients often experiencing symptoms such as depressed mood, fatigue, anxiety, social isolation, and reduced exercise capacity [1, 2].

Growth hormone (GH) is involved in the regulation of glucose levels, and impaired glucose metabolism, insulin resistance, and fasting hyperinsulinemia have been reported in adult patients with GHD [4, 5]. The increased abdominal obesity seen in GHD patients is also a likely contributor to the reduced insulin sensitivity observed in some patients [1, 6]. Furthermore, the prevalence of diabetes mellitus in adult GHD patients is significantly increased compared with the general population [7], particularly in those with additional risk factors such as a family history of diabetes mellitus or obesity [8].

GH replacement therapy aims to correct the metabolic, functional and psychological abnormalities that are associated with GHD [1, 9, 10]. Treatment with recombinant human GH (rhGH) has proved to be effective for improving body composition (increased lean body mass and decreased fat mass), exercise capacity, blood lipid profile and overall QoL in adult GHD patients [10]. In the early studies of GH replacement therapy, rhGH was administered intramuscularly 3 times per week in relatively high doses, adjusted according to patient body weight. In such studies, a further decrease in insulin sensitivity was reported after rhGH treatment initiation, which returned to baseline values over the first year of therapy [5]. However, since the 1990s rhGH has been administered subcutaneously once daily (guided by gender- and age-matched levels of insulin-like growth factor-I [IGF-I]), resulting in lower doses and fewer side effects. Moreover, insulin sensitivity and insulin resistance are reported to remain unchanged during long-term, low-dose rhGH treatment [5].

Most studies of GH replacement therapy in adults have indicated that glycated hemoglobin levels (HbA1c) remain stable, although a mild decrease was observed in one study over 15 years of GH treatment [11]. Monitoring of HbA1c is therefore recommended in adult GHD patients with additional diabetes mellitus risk factors (and in patients who already have diabetes mellitus), with adjustment of hypoglycemic medicines if necessary [8, 10].

Omnitrope® (somatropin; Sandoz) is a biosimilar rhGH approved by the European Medicines Agency in 2006, based on comparable quality, safety and efficacy to the reference medicine, Genotropin® (Pfizer) [12]. Omnitrope® is licensed to treat pronounced adult GHD of childhood or adulthood onset [13]. PAtients TReated with Omnitrope® (PATRO) Adults is an ongoing post-marketing surveillance study conducted in hospitals and specialized endocrinology clinics across Europe [12]. As Omnitrope® was approved in Europe as a biosimilar rhGH, the study is important to confirm that its long-term safety profile in adults with GHD is comparable to that of the reference medicine. The primary objective of PATRO Adults is to assess the long-term safety of Omnitrope® in adults with GHD treated in routine clinical practice. Secondary objectives include monitoring effectiveness parameters, including IGF-I levels, lipid profile and body composition. This paper presents data from PATRO Adults on the impact of rhGH therapy on glucose metabolism and the onset of diabetes mellitus in adult patients with GHD (data cut-off July 2018).

## Methods

PATRO Adults is an observational, multicenter, longitudinal, open-label, non-interventional study being conducted in hospitals and specialized endocrinology clinics across several European countries; patient recruitment began in 2007. The study design has been described in detail previously [12]. Briefly, eligible patients are male and female adults with GHD receiving Omnitrope® treatment in accordance with the recommendations in the Summary of Product Characteristics [13], and who have provided informed consent. Patients who received treatment with other rhGH medicines before starting Omnitrope® therapy are also eligible for inclusion [12].

The PATRO Adults study protocol was approved by the ethics review committee of participating centers in accordance with national laws and regulations. All procedures performed were in accordance with the ethical standards of these committees and with the 1964 Declaration of Helsinki and its later amendments.

All clinic visits and assessments are conducted as part of routine clinical practice according to the prescribing physician’s preference, with data collected at each routine visit during Omnitrope® treatment. Safety assessments include monitoring and recording of all adverse events (AEs), including AEs considered serious (SAEs) according to the definition provided in the International Conference on Harmonisation Guideline for Good Clinical Practice [14]. The relationship of AEs to rhGH treatment is made according to Investigator and Sponsor assessment (worst case). Particular emphasis is placed on the recording of incidence, severity and duration of hyperglycemia or diabetes mellitus during treatment with Omnitrope®.

The current interim analysis was performed in July 2018. All enrolled patients who have received at least one dose of Omnitrope® are included in the safety population. Patients without a recorded visit date or Omnitrope® treatment start date are excluded from the safety population. Standard descriptive statistics (mean, standard deviation, and frequency) are used to describe continuous parameters (e.g. age, height, weight) and categorical parameters (e.g. gender). Patient profiles, containing all available study database information for each specific patient, were generated for all patients with AEs of diabetes mellitus while participating in the study. Diabetes mellitus was confirmed if fasting plasma glucose was ≥7.0 mmol/L or 2-h plasma glucose ≥11.1 mmol/L during oral glucose tolerance test or HbA1c ≥6.5%. Diabetes mellitus cases were listed as AEs under the Medical Dictionary for Regulatory Affairs (MedDRA) High Level Term ‘Diabetes mellitus (including subtypes)’.

## Results

### Patient characteristics

As of July 2018, 1293 patients were enrolled into the PATRO Adults study from 76 centers in 8 European countries (Czech Republic, France, Germany, Italy, Netherlands, Spain, Sweden, UK). At the time of the analysis, 983 (76.0%) patients remained active in the study and 310 (24.0%) had discontinued. Baseline characteristics were generally similar between patients who remained active and those who had discontinued, although discontinued patients were slightly older (mean age 51.88 years vs 48.51 years) and the proportion of rhGH treatment-naïve patients was slightly higher (54.8% vs 47.5%). The majority of patients had multiple pituitary hormone deficiency (MPHD; *n =* 1128, 87.2%), with isolated GHD (*n =* 155, 12.0%) or other indications (*n =* 10, 0.8%) accounting for the remaining population (Table 1). Overall, 203 (15.7%) patients had childhood-onset GHD and 1080 (83.5%) patients had adult-onset GHD; information on time of onset was not available for 10 patients. Prior to enrolment into the study, 50.7% of patients had previously been treated with another rhGH and 49.3% of patients were rhGH-naïve. The mean (± standard deviation [SD]) body mass index (BMI) at baseline was 29.4 (± 6.4) kg/m^2^. Baseline BMI was < 18.5 kg/m^2^ in 14 (1.1%) patients, 18.5 to < 24.9 kg/m^2^ in 245 (18.9%) patients, 25.0 to < 29.9 kg/m^2^ in 412 (31.9%) patients, 30.0 to 34.9 kg/m^2^ in 233 (18.0%) patients, 35.0 to 39.9 kg/m^2^ in 107 (8.3%) and ≥ 40 kg/m^2^ in 81 (6.3%) patients; baseline BMI data were missing for 201 patients.

**Table 1:** – Patient characteristics at enrolment (safety population, *n =* 1293)

Indication	Pretreatment	Total, *n* (%)	Male, *n* (%)	Female, *n* (%)	Mean age (± SD) years	Mean BMI (± SD)^a^ kg/m^2^
Isolated GHD	Naïve	94 (7.3)	39 (3.0)	55 (4.3)	46.9 (15.4)	29.7 (6.6)
	Pretreated	61 (4.7)	27 (2.1)	34 (2.6)	42.7 (16.2)	31.3 (8.8)
MPHD	Naïve	537 (41.5)	289 (22.4)	248 (19.2)	49.5 (14.7)	29.7 (6.3)
	Pretreated	591 (45.7)	305 (23.6)	286 (22.1)	50.4 (15.6)	29.0 (6.2)
Other	Naïve	6 (0.5)	4 (0.3)	2 (0.2)	44.9 (13.1)	29.0 (0.6)
	Pretreated	4 (0.3)	2 (0.2)	2 (0.2)	31.7 (9.1)	26.0 (5.7)
Total		1293 (100.0)	666 (51.5)	627 (48.5)	49.3 (15.3)	29.4 (6.4)

*BMI* body mass index; *GHD* growth hormone deficiency; *MPHD* multiple pituitary hormone deficiency; *SD* standard deviation

^a^*n =* 1092 patients with BMI recorded at enrolment

Among patients who had discontinued the study (*n =* 310), 71 (5.5%) did so due to an AE. The intensity of AEs leading to discontinuation was reported as mild, moderate or severe in 10, 25 and 34 patients, respectively (missing data, *n =* 2). Other reasons for discontinuation included the patient not wanting to continue with injections (*n =* 66, 5.1%), patient lost to follow-up (*n =* 32, 2.5%), switch to a different rhGH treatment (*n =* 24, 1.9%), referral to another endocrinologist (*n =* 16, 1.2%), patient non-compliant (*n =* 10, 0.8%), and other (*n =* 91, 7.0%).

### Treatment

The median (range) duration of any rhGH pretreatment was 9.5 (0–42) years for patients with MPHD and 7.9 (1–32) years for those with isolated GHD. The median (range) duration of Omnitrope® treatment in the study was 37.3 (0–134) months and 37.1 (0–112) months for patients with MPHD and isolated GHD, respectively.

For patients with MPHD, the mean (± SD) Omnitrope® dose at baseline was 0.29 (± 0.22) mg/day. When split by pretreatment, the mean (± SD) dose was 0.20 (± 0.10) mg/day in naïve patients (rhGH starting dose) and 0.37 (± 0.26) mg/day in pretreated patients. Mean (± SD) dose was higher in childhood-onset MPHD patients compared with adulthood-onset GHD (0.41 [± 0.28] versus 0.26 [± 0.19] mg/day).

The mean (± SD) baseline dose for isolated GHD patients was 0.31 (± 0.27) mg/day. In rhGH-naïve patients, the mean (± SD) dose was 0.20 (± 0.14) mg/day (rhGH starting dose) versus 0.47 (± 0.33) mg/day in pretreated patients. Mean (± SD) dose was also higher in isolated GHD patients with childhood onset, compared with adulthood onset (0.47 [± 0.34] versus 0.26 [± 0.22] mg/day).

### Overall safety

Overall, 3828 AEs were reported in 872 (67.4%) patients; 153 AEs in 92 (7.1%) patients were suspected as related to study drug. Most AEs (90.3%; *n =* 3458) were mild to moderate in intensity, and the majority (91.3%; *n =* 3494) did not result in any change to Omnitrope® treatment. A total of 702 AEs in 353 (27.3%) patients were regarded as serious; 23 serious AEs in 18 (1.4%) patients were suspected as related to study drug.

### Occurrence of diabetes mellitus

Of the 1293 patients enrolled in PATRO Adults to date, 21 (11 male, 10 female) reported diabetes mellitus/inadequate control (worsening) of diabetes mellitus (*n =* 22 events; Table 2). The cases were newly diagnosed in 15 patients (age 29–84 years; incidence rate of 3.61 per 1000 patient-years) and occurred in 6 patients with pre-existing diabetes mellitus at baseline (age 45–72 years). Most cases of newly diagnosed diabetes mellitus occurred in patients with adult-onset MPHD (13 patients; 7 were rhGH-naïve and 6 were pretreated); the remaining cases of new-onset diabetes mellitus occurred in a patient with childhood-onset MPHD who had previously received GH replacement therapy (*n =* 1), and a patient with adulthood-onset isolated GHD who was naïve to GH replacement therapy (*n =* 1). All cases of inadequate control/worsening of diabetes mellitus occurred in patients with adult-onset MPHD (4 were treatment-naïve and 2 were pretreated)*.* Across all 21 patients with new-onset or worsening of diabetes mellitus, 2 had Cushing’s disease in their medical history and 14 were receiving cortisone replacement.

**Table 2:** – Adverse events and serious adverse events relating to diabetes mellitus

MedDRA PT (AE/SAE)	Indication and GHD onset, pretreatment status	Body weight at baseline (kg)	HbA1c at baseline (%)	Dose at AE onset^a^ (mg/day)	Time to AE/SAE onset^b^ (day)	BMI at AE onset (kg/m^2^)	Intensity	Causality^c^	Outcome	Treatment with Omnitrope®	Relevant medical history; Concomitant medication; Cardiovascular AE (Y if reported)
Diabetes mellitus (AE)	MPHD in childhood, pretreated	125.6	6.00	0.40	398	41.9	NR	Not suspected	Ongoing	Not changed	None
Diabetes mellitus (AE)	Isolated GHD in adulthood, naïve	67.4	6.08	0.20	694	28.1	Moderate	Not suspected	Ongoing	Not changed	None
Diabetes mellitus (SAE)	MPHD in adulthood, naïve	131.0	5.00	0.40	608	42.8	Moderate	Not suspected	Ongoing	Not changed	None; Hydrocortisone, statin
Diabetes mellitus inadequate control (AE)	MPHD in adulthood, naïve	131.0	5.00	0.40	2338	46.7	NR	Not suspected	Ongoing	Not changed	None; Hydrocortisone, statin
Diabetes mellitus [verbatim; worsening of diabetes] (SAE)	MPHD in adulthood, naïve	92.9	NR	0.20	127	29.3	Mild	Suspected	Ongoing	Permanently discontinued	Diabetes mellitus
Diabetes mellitus [verbatim; worsening of diabetes] (SAE)	MPHD in adulthood, pretreated	63.6	10.11	0.10	1	25.2	Mild	Suspected	Ongoing	Interrupted	Type 2 diabetes mellitus; Hydrocortisone, statin
Diabetes mellitus inadequate control (AE)	MPHD in adulthood, naïve	107.2	6.90	0.20	168	38.0	Mild	Not suspected	Ongoing	Not changed	Type 2 diabetes mellitus; Hydrocortisone, statin
Diabetes mellitus type 2 inadequate control (AE)	MPHD in adulthood, naïve	74.3	8.92	NR	NR	NR	Moderate	Not suspected	Ongoing	Not changed	Type 2 diabetes mellitus; Hydrocortisone, statin
Type 2 diabetes mellitus (AE)	MPHD in adulthood, naïve	107.6	6.00	0.15	841	39.6	Mild	Not suspected	Ongoing	Not changed	None; Hydrocortisone, statin
Type 2 diabetes mellitus (AE)	MPHD in adulthood, pretreated	99.7	6.18	0.50	1041	33.4	Moderate	Not suspected	Ongoing	Not changed	Cushing’s syndrome; Statin; Y
Type 2 diabetes mellitus (AE)	MPHD in adulthood, pretreated	95.8	NR	0.15	1130	37.9	Mild	Not suspected	Ongoing	Not changed	None; Hydrocortisone
Type 2 diabetes mellitus (AE)	MPHD in adulthood, pretreated	123.9	6.70	0.50	372	51.6	Mild	Not suspected	Ongoing	Not changed	Diabetes mellitus; Cushing’s syndrome; hydrocortisone
Type 2 diabetes mellitus (AE)	MPHD in adulthood, naïve	90.0	6.30	0.20	360	37.5	Mild	Not suspected	Ongoing	Not changed	Type 2 diabetes mellitus
Type 2 diabetes mellitus (AE)	MPHD in adulthood, naïve	111.3	6.00	0.14	650	36.2	Mild	Not suspected	Resolved completely	Not changed	None; Cortisone acetate, statin
Type 2 diabetes mellitus (AE)	MPHD in adulthood, pretreated	94.0	NR	0.50	998	27.8	Mild	Not suspected	Ongoing	Not changed	None
Type 2 diabetes mellitus (SAE)	MPHD in adulthood, pretreated	108.1	5.90	0.30	955	43.2	Mild	Not suspected	Ongoing	Not changed	None; Hydrocortisone, statin
Type 2 diabetes mellitus (SAE)	MPHD in adulthood, pretreated	104.6	NR	NR	NR	NR	Mild	Not suspected	Ongoing	Not changed	None; Hydrocortisone
Type 2 diabetes mellitus (SAE)	MPHD in adulthood, naïve	106.7	NR	0.30	9	39.2	Mild	Not suspected	Ongoing	Not changed	None; Statin
Type 2 diabetes mellitus (SAE)	MPHD in adulthood, naïve	105.0	5.99	0.40	521	32.3	Mild	Not suspected	Ongoing	Not changed	None; Hydrocortisone, statin
Type 2 diabetes mellitus (SAE)	MPHD in adulthood, pretreated	63.7	5.90	0.20	1274	26.1	Moderate	Not suspected	Ongoing	Not changed	None; Hydrocortisone, statin; Y
Type 2 diabetes mellitus (SAE)	MPHD in adulthood, naïve	95.0	NR	0.36	1317	32.5	Mild	Not suspected	Ongoing	Not changed	None; Cortisone acetate, statin; Y
Type 2 diabetes mellitus (SAE)	MPHD in adulthood, naïve	100.0	6.91	0.30	260	33.8	Mild	Not suspected	Ongoing	Not changed	None; Hydrocortisone

^a^Last documented dose before AE onset in mg/day; ^b^Time to SAE onset after start of Omnitrope® treatment; ^c^Assessment of relationship to study drug according to Investigator and Sponsor (worst case)

*AE* adverse event; *BMI* body mass index; *GHD* growth hormone deficiency; *HbA1c* glycated hemoglobin; *MedDRA* Medical Dictionary for Regulatory Activities; *MPHD* multiple pituitary hormone deficiency; *NR* not recorded; *PT* Preferred Term; *SAE* serious adverse event; *Y* Yes

Diabetes mellitus was recorded as a non-serious AE in 12 patients (*n =* 12 events), and these AEs were mild to moderate in intensity (excluding 2 patients with missing severity data). None of these AEs were considered to be related to rhGH treatment. All diabetes mellitus AEs were ongoing, except for a single patient for whom complete resolution of their diabetes mellitus was recorded (Table 2). No change to the Omnitrope® treatment regimen was required in these 12 patients.

Diabetes mellitus was recorded as an SAE in 10 patients (*n =* 10 events), all of which were ongoing at the time of analysis. In two of these patients, the SAE was considered possibly related to rhGH treatment (MedDRA preferred term *diabetes mellitus*; verbatim term *worsening of diabetes,* for both patients). The first of the treatment-related cases of diabetes mellitus was in a male patient with adulthood-onset MPHD, who was previously naïve to rhGH therapy. Diabetes mellitus was a pre-existing condition at baseline in this patient. Worsening of the patient’s diabetes mellitus was reported on Day 127 of Omnitrope® treatment. The SAE was recorded as mild in intensity but required permanent discontinuation of rhGH treatment. The second case of treatment-related diabetes mellitus was reported in a male patient with adult-onset MPHD and pre-existing type 2 diabetes mellitus. This patient was previously treated for approximately 18.6 years with rhGH before starting Omnitrope® treatment (previous rhGH treatment stopped in 2011, Omnitrope treatment started in January 2014). The SAE was reported on Day 1 of Omnitrope® treatment, was considered mild in intensity but the patient requested rhGH treatment to be interrupted (Table 2). Further follow-up identified that treatment had been permanently discontinued and that diabetes mellitus was reported as ‘condition improving’.

### Glycated hemoglobin

**Fig. 1:**
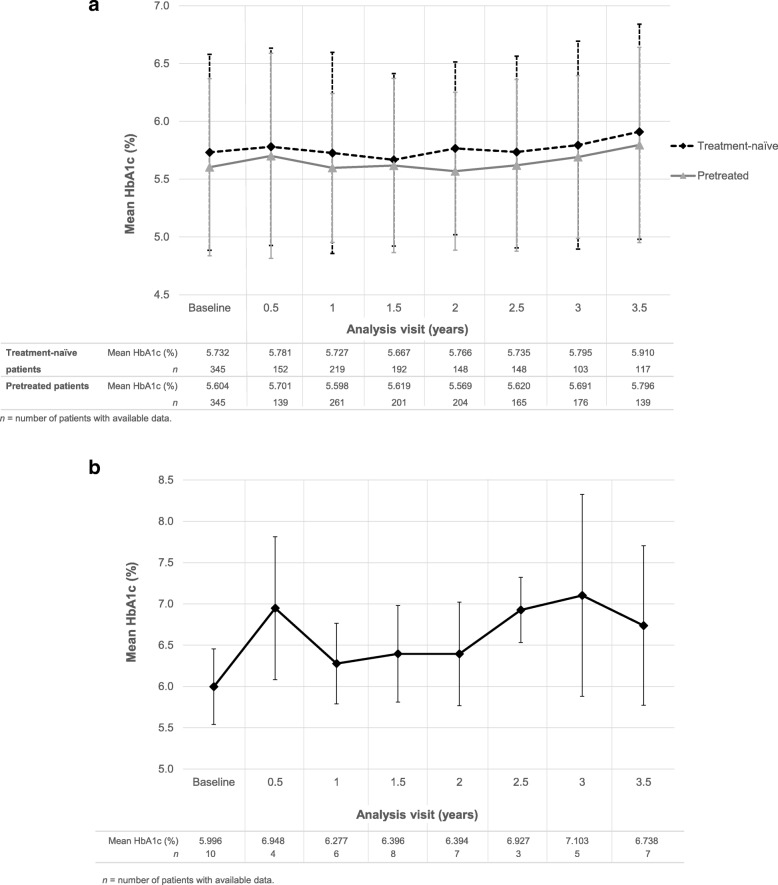
– HbA1c (± SD) over 3.5 years in (**a**) treatment-naïve and pretreated adult GHD patients and (**b**) patients who developed new-onset diabetes mellitus (safety population)

Overall, HbA1c levels remained stable during treatment with Omnitrope® (Fig. 1a). At 3.5 years, the mean (± SD) change in HbA1c (%) from baseline was + 0.277 (± 0.528) in treatment-naïve patients and + 0.154 (± 0.714) in pretreated patients. Figure 1b shows HbA1c levels over time in those patients who had diabetes mellitus newly diagnosed during the study.

## Discussion

The PATRO Adults post-marketing surveillance study is designed to gather valuable data on the long-term safety of Omnitrope® treatment in adult patients with GHD in a real-life clinical setting. The current analysis indicates that Omnitrope® treatment in adult patients with GHD does not markedly increase the risk of diabetes mellitus and glucose metabolism disorders in this population. These findings are in line with other published data of rhGH treatment in adult GHD patients. The observational Kabi Pharmacia International Metabolic Study (KIMS) included 5143 adult patients with GHD receiving GH replacement therapy, with 20,106 patient-years of follow-up. In this study, the diabetes mellitus incidence was 2.6 per 100 patient-years, gradually decreasing from a peak of 4.1 per 100 patient-years in year 2 of GH replacement therapy to 1.0 per 100 patient-years after > 8 years of treatment, irrespective of gender. When diabetes mellitus incidence rates were compared with those of 4 age-matched populations from southern/central Europe and the USA, the observed-to-expected case ratios ranged from 2.11 to 5.22. Furthermore, the patients who developed diabetes mellitus were found to have additional risk factors (such as higher BMI, waist circumference, triglyceride concentrations, and blood pressure) compared with patients who did not develop diabetes mellitus [15].

An analysis of the Hypopituitary Control and Complications Study (HypoCCS; Eli Lilly) included 2922 adult GHD patients from the USA and 3709 from Europe, with a mean follow-up period of 4.1 years. The overall incidence of diabetes mellitus (adjusted for age, gender and BMI) was 9.7 per 1000 patient-years (14.1 and 7.0 per 1000 patient-years in the US and Europe, respectively). The diabetes mellitus incidence rate in patients from the USA and Sweden was higher compared with the untreated reference populations, but in patients from France and Germany the incidence rates were similar to the reference populations [16]. Furthermore, for patients with BMI < 25 kg/m^2^, the diabetes mellitus incidence was 2.1 per 1000 patient-years, which increased to 16.4 per 1000 patient-years for patients with a BMI over 30 kg/m^2^. These findings suggest that the abdominal obesity often observed in adult GHD patients may be linked to the higher rate of diabetes mellitus reported, rather than the rhGH treatment itself [16].

The NordiNet® non-interventional study of non-diabetic patients with adulthood-onset GHD (*n =* 245) showed no adverse effects of > 4 years of GH replacement therapy on glucose homeostasis in the majority of patients. In this study, 7 patients developed diabetes mellitus, but they tended to have additional risk factors compared with the rest of the study population, including older age, higher BMI at baseline, and concomitant glucocorticoids [17]. However, a meta-analysis of 94 studies did not demonstrate an increased frequency of diabetes mellitus in short-term, placebo-controlled trials, nor was the incidence of diabetes mellitus increased during long-term GH replacement studies [18].

As gluconeogenesis and glycogenolysis are physiological effects of GH therapy, an increase in glucose levels within the normal range may be expected during rhGH treatment. Stable levels of HbA1c were observed in the present study, consistent with most studies of GH replacement in adults; a mild decrease was observed in one study of long-term (15 years) of GH treatment [11]. A review of data from large-scale registry studies of patients receiving GH replacement therapy concluded that the incidence of diabetes mellitus may only be slightly increased in patients with pre-existing risk factors for diabetes mellitus, rather than due to changes resulting from rhGH therapy [19]. Consistent with this, risk factors (e.g.*,* overweight, Cushing’s disease) were noted in patients who developed diabetes mellitus/worsening of diabetes mellitus in the present study. Monitoring of HbA1c is recommended in adult GHD patients who have risk factors for diabetes mellitus (or pre-existing diabetes) and receive GH replacement, with adjustment of hypoglycemic medications if needed [8, 10].

Nineteen of the 21 patients who developed or had worsening of diabetes mellitus had adult-onset MPHD, and most of these patients were receiving concomitant cortisone (*n* = 14 patients) or statins (*n* = 12 patients). Cortisone and other hormone replacement therapies have their own effects on the metabolic profile. Cortisone therapy has been linked to the increased incidence of diabetes and impaired glucose tolerance observed in patients with hypopituitarism, although a lower prevalence is reported in patients receiving smaller cortisone doses [20]. Similarly, the use of statins has been associated with an increased prevalence of new-onset diabetes mellitus [21]. The relationship between the development of diabetes and cortisone or statin use could not be confirmed in our analysis, as precise information on the dosing of these medicines could not be retrieved from the study database. Further investigation into the effect of cortisone and statin use in patients receiving rhGH therapy is therefore warranted. It is recommended that patients receiving rhGH therapy, particularly those with risk factors for diabetes mellitus, are monitored closely for glucose level impairment [3, 9, 10].

The PATRO Adults study is expected to provide additional information on the risk of increased insulin resistance and changes in glucose tolerance, due to its longitudinal design and large sample size. However, the study has some limitations, which are common to all observational studies. Firstly, there is a potential selection bias due to the inclusion of selected clinics and enrolment of patients from only these clinics. As data is collected according to routine clinical practice in the study, there is potential for information bias (due to incorrect or inexact recording of information) and the amount of data available for some analyses is low. Also, the PATRO Adults database does not contain information on diet and levels of physical activity (or any changes in these). Due to the observational nature of the study, the choice of method used to diagnose diabetes (OGTT or HbA1c) was not recorded for all patients and likely reflects the reference of the individual clinician; a bias in the rate of diabetes diagnosis is therefore possible. Furthermore, the relatively small sample size may limit the interpretation of some data. Finally, there is often a long time period between patient visits in the PATRO Adults study (6–12 months) and limited consultation time during routine visits, which may lead to under-reporting of AEs.

## Conclusions

The latest data from PATRO Adults confirm that Omnitrope® treatment is tolerated in adult patients with GHD in a real-life clinical practice setting. Consistent with previous experience with rhGH, there have been no signals of an increased risk of diabetes mellitus and glucose metabolism disorders associated with Omnitrope®. Nevertheless, close glucose monitoring is required in all patients receiving rhGH treatment. PATRO Adults will continue to provide valuable data on the long-term safety of Omnitrope® in adults with GHD, as well as contribute to the safety profile for all rhGH medicines.

## Data Availability

The datasets generated during and/or analyzed during the current study are not publicly available as the study is still ongoing, but are available from the corresponding author on reasonable request.

## Abbreviations

AE: Adverse event
BMI: Body mass index
GH: Growth hormone
GHD: Growth hormone deficiency
HbA1c: Glycated hemoglobin
HypoCCS: Hypopituitary Control and Complications Study
IGF-I: Insulin-like growth factor-I
KIMS: Kabi Pharmacia International Metabolic Study
MedDRA: Medical Dictionary for Regulatory Affairs
MPHD: Multiple pituitary hormone deficiency
PATRO: PAtients TReated with Omnitrope®
QoL: Quality of life
rhGH: Recombinant human growth hormone
SAE: Serious adverse event
SD: Standard deviation
